# The Relationships between Waxes and Storage Quality Indexes of Fruits of Three Plum Cultivars

**DOI:** 10.3390/foods12081717

**Published:** 2023-04-20

**Authors:** Shouliang Zhu, Shian Huang, Xin Lin, Xuan Wan, Qin Zhang, Junsen Peng, Dengcan Luo, Yun Zhang, Xiaoqing Dong

**Affiliations:** 1 Guizhou Workstation for Fruit and Vegetables, Guiyang 550025, China; 2 Fruit Crops Center of Guizhou Engineering Research, College of Agricultural, Guizhou University, Guiyang 550025, China; 3 Guiyang Agricultural Reclamation Investment Development Group Co., Ltd., Guiyang 550001, China

**Keywords:** plum fruit, cuticular wax, wax structure, chemical composition, storage quality, correlation analysis

## Abstract

In the present study, the cuticular wax morphology, composition and the relationship with storage quality in three plum cultivars of *Prunus salicina* ‘Kongxin’ (KXL), *Prunus salicina* ‘Fengtang’ (FTL) and *Prunus salicina* ‘Cuihong’ (CHL) were investigated during storage at room temperature of 25 ± 1 °C. The results illustrated that the highest cuticular wax concentration was discovered in KXL, followed by FTL and the lowest in CHL. The fruit wax composition of the three plum cultivars was similar and principally composed of alkanes, alcohols, fatty acids, ketones, aldehydes, esters, triterpenes and olefins. Alcohols, alkanes and triterpenes were the dominant fruit wax compounds of the three plum cultivars. After storage for 20 d at room temperature, the variation of cuticular wax crystal structure and composition showed significant cultivar-associated differences. The total wax content decreased for FTL and CHL and increased for KXL, and the wax crystal degraded and melted together over time. The higher contents of the main components in the three plum cultivars were nonacosane, 1-triacontanol, 1-heneicosanol, nonacosan-10-one, octacosanal, ursolic aldehyde and oleic acid. Alcohols, triterpenes, fatty acids and aldehydes were most dramatically correlated with the softening of fruit and storage quality, and alkanes, esters and olefins were most significantly correlated with the water loss. Nonacosane and ursolic aldehyde can enhance the water retention of fruit. Overall, this study will provide a theoretical reference for the further precise development of edible plum fruit wax.

## 1. Introduction

A great number of fruits, including plum, develop a pronounced, conspicuous layer of epicuticular wax responsible for their attractive visual appearance and protective function. Waxes secreted from the epidermis of fruits are less soluble in water and more soluble in organic solvents during fruit development and storage [1], and these layers of metabolic substances are the first barriers of interaction between fruits and the external environment [2].

The hydrophobic wax effectively prevents the fruit from non-stomatal water loss [3,4], promotes the waterproof characteristics of the fruit skin, keeps the skin surface clean [5] and can significantly contribute to disease and insect pest resistance [6,7] and prevent the fruit from cracking [8,9]. Waxes can effectively prevent loss and shrinkage during storage [10,11,12], regulate physiological disorders [13] and reduce the incidence of fruit diseases [14], thereby ensuring quality and commercial value of the fruit [15]. For example, cuticular wax delayed the softening of blueberry [16], tomato [17], melon [13] and grape [5] and reduced chilling injury of papaya [18].

Cuticular wax is a mixture of hydrophobic lipids, including fatty acids, alkanes, alcohols, aldehydes, ketones, esters and triterpenes, which covers the outermost layers of fruits, flowers, stems and leaves of several plants [6,19]. The total wax amount and the chemical composition vary depending on species, cultivar, development stage and storage conditions [20,21,22] and play important roles in preservation of the postharvest quality. The retention of natural wax maintains the postharvest quality of blueberry fruit and delays its senescence [10]. The total wax content in apple peel, particularly alkanes, was essential for fruit storage and quality control [12]. The total wax load and wax composition of olefins and fatty acids followed by alkanes and esters in Korla pear fruit were closely related to weight loss and postharvest senescence [11]. The triterpenes, mainly ursolic acid content, in cuticular wax affected the softening rates in blueberry [23].

Wax gives fruits a frosty white coating or colorless smooth appearance [2,5]. Wax bloom is an important sensory quality of plum fruit, and intact fruit wax without scratch enhances the freshness of plum fruit. However, freshly harvested plums exhibit rapid weight loss, which is a major concern for edible quality and marketing, as well as the main reason for their short storage life [24]. Although Mukhtar et al. [4] demonstrated that there was a sharp increase in postharvest water loss upon the removal of wax from the surface of European plum, little is known about wax structure, composition in different plum cultivars and relationship with storage quality. Therefore, it is necessary to investigate the morphology and composition of plums during postharvest storage and to understand the effect on the fruit quality in different cultivars.

*Prunus salicina* ‘Fengtang’ (FTL) is a local characteristic plum cultivar in Guizhou Province and is called ‘Fengtang’ plum due to its fragrant taste and sweet pulp as honey. It is a local excellent plum cultivar produced in Liuma Town, Zhenning County, Anshun City, Guizhou Province [25]. *Prunus salicina* ‘Kongxin’ (KXL) is named after the natural division of the fruit stone and flesh at maturity and enjoys the reputation of ‘Maotai in plum’ and is mainly cultivated in Yanhe County, Tongren City, Guizhou Province [26]. *Prunus salicina* ‘Cuihong’ (CHL) is a late-mature variety selected from the fruiting offspring of Chinese prunus which is named for its red skin and crisp, sweet flesh and is primarily planted in Sichuan, Chongqing, Guizhou and other southwest regions [27].

A large number of studies have illustrated that cuticular wax morphology properties as well as composition and content of wax of fruits such as apples [12], blueberries [16], grapes [5] and pears [28] are the predominant factors that determine the postharvest storage quality and life. Nevertheless, available information about wax structure, composition in different cultivars and relationship with storage quality are limited. Therefore, the present research was performed to investigate the cuticular wax and storage quality variations among three plum cultivars and further examine the potential relationships between cuticular wax and postharvest quality parameters and provide a theoretical reference for the precise development of edible plum fruit wax.

## 2. Materials and Methods

### 2.1. Plant Materials

The fruit of KXL, FTL and CHL with similar shape and size and no damage were selected as the research objects. Among them, KXL plum, with a firmness of approximately 5.5–7.0 N and total soluble solids (TSS) of approximately 8.7–10.6%, was picked from an orchard (108.49° E, 28.56° N) of a farmers’ professional cooperative of Yanhe Tujia Autonomous County, Guizhou Province. FTL plum, with a firmness of approximately 10.2–11.7 N and TSS of approximately 13.3–15.5%, was harvested from an orchard (105.89° E, 25.67° N) of a farmers’ professional cooperative of Liuma town, Guizhou Province. CHL plum, with a firmness of approximately 19.8–12.0 N and TSS of approximately 9.4–10.1%, was picked from the experimental base of Fruit Research Institute, Guizhou Academy of Agricultural Sciences (108.32 °E, 28.32° N) in the year of 2021. Polyvinyl chloride gloves were chosen for manual picking. Fruit was gently picked and placed with minimal disturbance of the wax bloom. After picking, the fruits were placed in a plastic basket (540 × 360 × 310 mm) and transported to the laboratory for treatment within 4 h after picking, precooled for 12 h (25 ± 1 °C) and stored at room temperature (25 ± 1 °C, 85 ± 1% relative humidity).

### 2.2. Scanning Electron Microscopy of Plum Cuticular Wax

Scanning electron microscopy was employed to observe microstructural changes in the plum epidermis wax. Each fruit was sampled at three points at the equator (directly opposite the fruit umbilical, on both sides) to observe the wax structure of the fruit and cut into strips (3 × 3 × 2 mm), fixed in 2.5% (*w*/*v*) glutaraldehyde solution and stored at 4 °C for 12 h. Next, the strips were soaked with 2% phosphate buffer (pH = 7.0) for 3 min. Then, gradient tert-butyl alcohol (50%, 70%, 90%) was used for dehydration once, 10 min for each time. Finally, 100% tert-butyl alcohol was used for dehydration twice, 10 min for each time. After dehydration treatment, the strips were vacuum-pumped and dried in freeze dryer (LGJ-10D, Beijing China). Subsequently, the samples to be observed were adhered to the sample plates of the scanning electron microscope, and gold was sputter-sprayed by an ion sputter coater (E-1010, HITACHI, Tokyo, Japan). Samples were subjected to observations at magnifications of 300 and 2000 times with a scanning electron microscope (S3400N, HITACHI, Japan).

### 2.3. Cuticular Wax Extraction

Cuticular wax extraction was performed using the method proposed by Lin et al. [26]. Cuticular wax was extracted from three varieties of plum fruits stored for 0, 10 and 20 d. Fruits of the same shape and size (*n* = 10) were selected, and each fruit was extracted in three 500 mL beakers for 30 s in turn so that all the fruits were immersed and agitated for 30 s in chloroform/methanol (*v*/*v*: 4/1) under a fume hood, which ensured that the fruit epidermis was not damaged. The extracted solution was mixed and filtered, concentrated at 40 °C by a rotary evaporator, then transferred to a pre-weighed 40 mL transparent brown glass screw bottle and dried to a constant mass under a dry nitrogen blower (MD200–2, Shanghai Huxi Industry Company Limited, China) at 40 °C (weight change of wax was no more than 10 mg within 30 min). After that, the wax was sealed and stored at −80 °C for further measurement. The transverse diameter (d1), longitudinal diameter (d2) and height (h) of extracted fruit were measured, and the surface area of a single fruit was determined as S = [4 × 3.14 × (d1 + d2 + h)^2^]/36. The cuticular wax content of fruit (μg·cm^−2^) was calculated as (W_1_ − W_0_)/St; W_1_ = final weight of screw bottle (μg); W_0_ = initial weight of screw bottle (μg); and St = total of ten fruit surface areas (cm^2^) [12].

### 2.4. Cuticular Wax GC-MS Analysis

The samples stored at −80 °C were redissolved in 10 mL chloroform followed by 1 min of vortex and 5 min of ultrasound. An aliquot (2.5 mL) of the redissolved solution was transferred to a 4 mL glass bottle, and wax extract was blown dry with liquid nitrogen. Chloroform (500 μL) and 80 μL N,O-bis (trimethylsilyl) trifluoroacetamide (BSTFA) reagent (containing 1% chlorotrime–thylsilane (TMCS)) were added to a 4 mL glass bottle filled with the nitrogen-dried sample. The samples were then vortexed for 1 min and placed in an ultrasound for 5 min, then reacted at 70 °C for 1 h; samples were placed at room temperature for 30 min followed by centrifugation at 12,000× *g* for 10 min. The supernatant (200 μL) was transferred to a glass bottle with liner for GC-MS metabolomic analysis. Mixed standard of n-alkanes (C7–C40, 20 μg·mL^−1^) was transferred to a glass bottle with a lined tube for GC-MS metabolomic analysis.

GC-MS analysis was carried out using a DB-5MS capillary column (30 m × 0.25 mm × 0.25 μm, Agilent J&W Scientific, Folsom, CA, USA) with ultra-pure helium (purity not less than 99.999%) as carrier gas at a flow rate of 1.0 mL·min^−1^ with the inlet temperature at 260 °C. The heating procedure was as follows: the initial temperature was set to 80 °C, rose to 200 °C at 10 °C·min^−1^ for 2 min, increased to 260 °C at 15 °C·min^−1^ and finally rose to 315 °C at 5 °C·min^−1^ for 10 min.

Mass spectrometry conditions were as follows: an electron bombardment ion source was used, ion source temperature was 230 °C, quadrupole temperature was 150 °C and electron energy was set to 70 eV. The scanning mode was full-scan mode (SCAN), and the quality scan ranged from *m*/*z* 50 to 650. The wax components were qualitatively analyzed using the NIST database (https://webbook.nist.gov/chemistry/ (accessed on 7 November 2022)) or by comparing the mass spectrum and retention time of the component substances with the retention time for qualitative determination, and quantification of the wax composition was determined by an internal standard with a known content. The components with similarity greater than 80 were selected as the final results [29].

### 2.5. Weight Loss

Ten fruits of each treatment of each cultivar with the same size were fixed to observe the fruit weight loss with the weighing method using the average value as the results. Weight loss (%) was calculated according to the formula (%) = [(*M*_0_ − *M*_1_)/*M*_0_] × 100, where *M*_0_ and *M*_1_ represent the initial weight of each plum and the measured weight of each plum during storage, respectively.

### 2.6. Fruit Firmness

Six fruits were randomly selected in each treatment, and two symmetrical locations at the equator of the fruit were peeled (about 1 mm thick) to determine the fruit firmness. Fruit firmness was measured using a digital fruit pressure tester (GY-4, Handpi Industry Company Limited, Wenzhou, China) with a 3.5 mm diameter cylindrical probe. Results were expressed in newtons (N).

### 2.7. Total Soluble Solids (TSS) and Titratable Acid (TA) Content

TSS and TA were measured using a handheld digital refractometer (Model PAL-BX/ACID1, Atago Co. Ltd., Tokyo, Japan) by dripping the fruit juice into the sample slot to determine the value of the TSS, followed by diluting the juice with distilled water 50 times to calculate the value of the TA. Values were expressed as percentages (%). The values of firmness, respiration rate, TSS and TA were determined in triplicate [25,26].

### 2.8. Soluble Sugar and Soluble Protein Content

The soluble sugar content was determined by anthrone colorimetry, and the results were expressed as mass fractions (%) [30]. The content of soluble protein was explored using Coomassie brilliant blue G-250 colorimetry, and the results were expressed as soluble protein content per gram of fruit (mg·g^−1^) [31].

### 2.9. Statistical Analysis

All experiments were performed in triplicate, and all data were analyzed using one-way analysis of variance (ANOVA) by employing the statistical software of SPSS 19.0 for Windows (SPSS Inc., Chicago, IL, USA). Duncan’s multiple range test (*p* ≤ 0.05) was employed to analyze the storage quality data, and Origin Pro 2021 was chosen to draw the histogram. The correlation plot of Origin Pro 2021 was used for the correlation heatmap. Data are shown as the means ± SE (*n* = 3).

## 3. Results

### 3.1. Changes in Appearance and Cuticular Wax Morphology of Three Plum Cultivars during Storage

**Figure 1:**
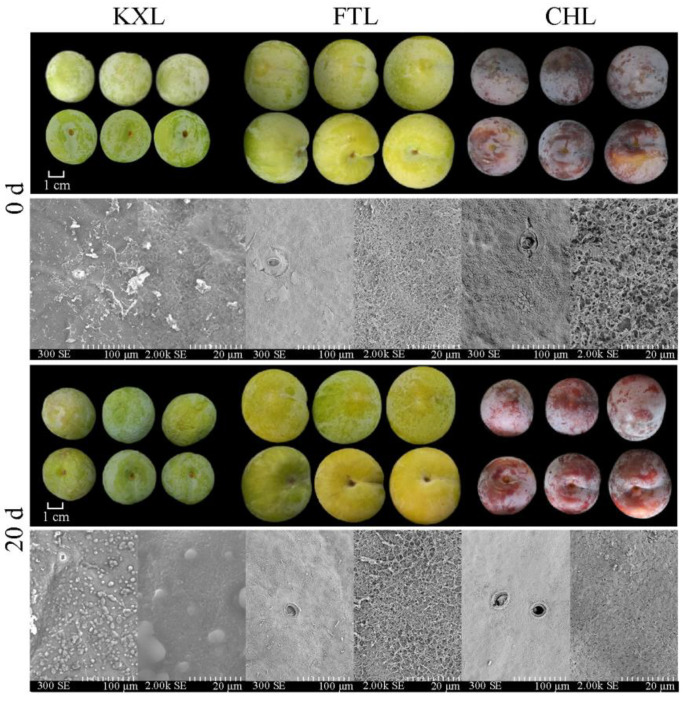
Changes in fruit appearance and cuticular wax structure among three plum cultivars during storage at room temperature. Note: the size of 300 SE was lenticels, and the size of 2.00 k SE was pericarp wax structure.

Peel colors of KXL, FTL and CHL were emerald-green, yellowish-green and rose-red at harvest time, respectively. After storage for 20 d at room temperature of 25 ± 1 °C, KXL fruit wrinkled seriously; however, the fruit powder remained intact and became heavier, and the peel deepened. FTL shrinkage was more serious than KXL, the fruit powder reduced and the fruit changed from yellowish-green to brownish yellow. CHL did not shrivel up, the fruit powder decreased less than that of FTL, the rose red of fruit peel deepened and the fruit gradually turned red (Figure 1).

At 0 d, the cuticular wax of KXL was complete and was a mixture of dense flaky wax as well as some rod-shaped wax, with a great variety of folds and small cracks on the wax. The cuticular wax integrity of FTL was lower than that of KXL, and its surface was relatively smooth, with a large amount of granule-shaped wax covered with platelet-shaped and rod-shaped wax crystals. The cuticular wax integrity of CHL was lower than that of FTL, and the surface of CHL was covered with a large number of cracks, which were composed of rod-shaped wax with large porosity, and the surface of the wax layer was randomly covered with a small number of platelet-shaped wax crystals. Moreover, the lenticels of KXL were smaller than those of FTL and CHL (Figure 1).

After 20 d storage at room temperature, the wax structure of the three plum fruits changed differently compared with that of 0 d. A great variety of oil-droplet wax structures were distributed on the peel of KXL, and the surface was rugged, the folds and cracks of peel wax disappeared and the oil-droplet wax collected around the lenticels, which was clearly exposed on the peel. The wax integrity of FTL decreased, the granule-shaped wax crystals covering the platelet-shaped wax almost disappeared, and the rod-shaped wax gradually decreased and adhered. The cutin around the lenticels thickened, and gaps around the lenticels were covered by wax. The wax integrity of CHL increased, and its surface became smooth, platelet-shaped wax crystals were more abundant and there were more small holes. The change of the lenticels was consistent with that of FTL (Figure 1).

### 3.2. Changes in Cuticular Wax Proportion and Content of Three Plum Cultivars during Storage

**Figure 2:**
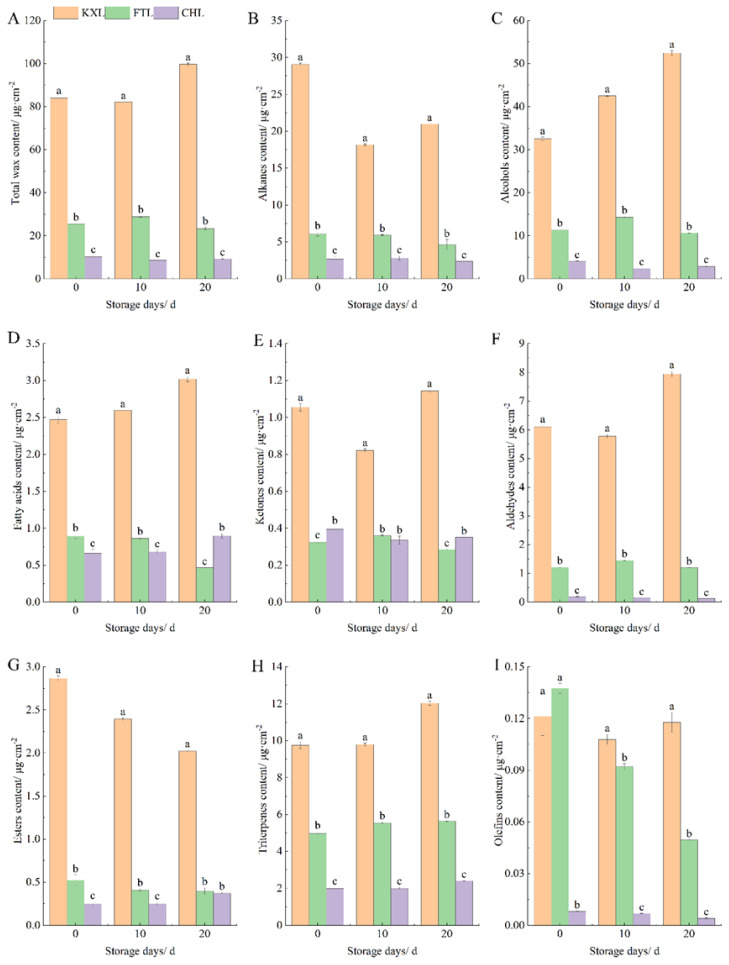
Changes in content of cuticular wax composition among three plum cultivars during storage at room temperature. (**A**), Total wax; (**B**), alkanes; (**C**), alcohols; (**D**), fatty acids; (**E**), ketones; (**F**), aldehydes; (**G**), esters; (**H**), triterpenes; (**I**), olefins. Note: vertical bars express the standard errors of the means (*n* = 3). Different lowercase letters indicate significant differences for each storage period (*p* < 0.05).

**Figure 3:**
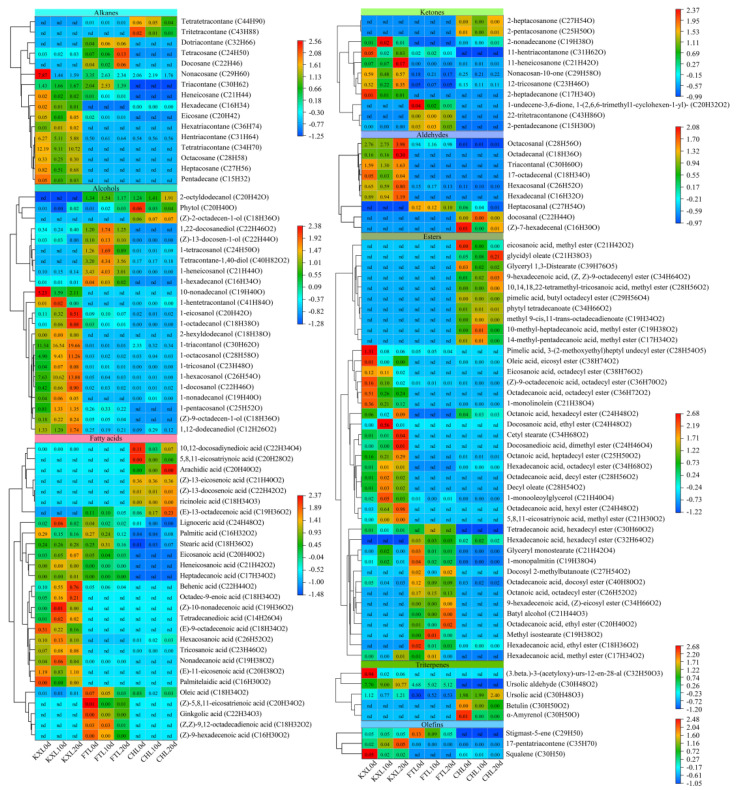
Change heatmaps of cuticular wax composition and concentration among three plum cultivars during storage at room temperature. Note: nd represents an undetected substance, and 0.00 means that the content of substance was lower than 0.01 μg·cm^−2^. Red and blue colors indicate high and low content, respectively. Each square represents the means of three replicates (*p* < 0.05).

Changes in the proportions and contents of cuticular wax composition are shown in Table 1 and Figure 2 and Figure 3. The results from GC-MS demonstrated that the wax composition of three plum cultivars was similar, primarily including 16 alkanes, 23 alcohols, 28 fatty acids, 11 ketones, 9 aldehydes, 40 esters, 5 triterpenes and 3 olefins. However, it was different for the proportions of different components of three plum cultivars during storage. At harvest time, alcohols, alkanes and triterpenes were the top three main fractions in KXL, FTL and CHL, followed by aldehydes (7.27%) and esters (3.41%) for KXL, aldehydes (4.76%) and fatty acids (3.50%) for FTL and fatty acids (6.5%) and ketones (3.87%) for CHL, respectively, while olefins had the lowest content (0.14%, 0.54% and 0.08%, respectively) (Table 1). The content of various cuticular wax components for total wax, alcohol, aldehyde, alkane, ester, fatty acid, ketone and triterpene of KXL was significantly higher than that of FTL and CHL, but the content of olefin of KXL was slightly lower than FTL at 0 d. FTL had higher content than CHL except ketones (Figure 2). After 20 d storage at room temperature, the species did not change; however, the content of each composition changed with different trends, and the proportion of wax in KXL was in the order alcohols > alkanes > triterpenes > aldehydes > fatty acids > esters > ketones > olefins. The proportion of wax in FTL was in the order alcohols > triterpenes > alkanes > aldehydes > fatty acids > esters > ketones > olefins. The proportion of wax in CHL was in the order alcohols > triterpenes > alkanes > fatty acids > esters > ketones > aldehydes > olefins.

#### 3.2.1. Total Wax Amounts

The total wax content in the epidermis of KXL, FTL and CHL was 83.96, 25.50 and 10.20 μg·cm^−2^, respectively, when harvested. The pattern of total wax changes of KXL was similar to CHL, as it decreased first and then increased during storage; however, it was different from FTL, which increased first and then decreased (Figure 2A). After storage for 20 d at room temperature, the total amounts of wax reached 99.67 (KXL), 23.35 (FTL) and 9.37 μg·cm^−2^ (CHL) and increased by 18.71% for KXL and decreased by 8.46% and 8.20% for FTL and CHL. Compared with KXL, the fluctuation of the wax amount in FTL and CHL changed more slightly.

#### 3.2.2. Alkanes

The alkanes content of KXL, FTL and CHL was 29.06, 6.08 and 2.68 μg·cm^−2^, accounting for 34.61%, 23.82% and 26.25% of total wax content, respectively, and decreased during storage (Figure 2B and Table 1). A total of 16 alkanes were detected, and tetratriacontane was the alkane with the highest content in KXL, accompanied by nonacosane and hentriacontane for KXL, which first showed an increasing and then decreasing tendency, but it was lower at 20 d than at 0 d (Figure 3 and Figure 2B). With prolonged storage time, each composition of KXL decreased, except triacontane and eicosane and hexatriacotane with a slight increase. The alkane with the highest content in FTL and CHL was nonacosane, and its content decreased during storage. Nonacosane had the second highest content of the alkanes in KXL (7.87 μg·cm^−2^), which was higher than in FTL (3.35 μg·cm^−2^) and CHL (2.06 μg·cm^−2^). Among those alkanes, only nonacosane and hentriacontane existed in three plum cultivars (Figure 3).

#### 3.2.3. Alcohols

Alcohol was the most abundant component in plum cuticular wax, accounting for 38.74%, 44.45% and 39.53% for KXL, FTL and CHL, respectively (Table 1). The content of alcohol in the three varieties of plum revealed different change trends. The alcohol content in KXL increased gradually during storage, and it increased initially and decreased subsequently for FTL; however, it decreased firstly and was slightly higher at 20 d than at 10 d for CHL (Figure 2C). Among the alcohols, the highest-abundance alcohol component in KXL was 1-triacontanol, which continued to increase from 11.34 to 19.66 μg·cm^−2^ during storage. For KXL, all components except 10-nonadecanol and (Z)-13-docosen-1-ol increased. The highest amount of alcohol in FTL was exhibited by 1-heneicosanol, followed by tetracontane-1,40-diol, which presented a similar change trend to the total alcohol. The highest content of alcohol in CHL was exhibited by 1-triacontanol at 0 d, was along with 2-octyldodecanol at 10 and 20 d. In addition, three diols, namely 1,22-docosanediol, tetracontane-1,40-diol and 1,12-dodecanediol, were identified in three plum cultivars, and 1,22-docosanediol was detected in KXL and FTL, 1,40-tetracontanediol in FTL and CHL and 1,12-dodecanediol in those three kinds of plums (Figure 3).

#### 3.2.4. Fatty Acids

The fatty acid content of FTL decreased continuously during storage from 0.89 to 0.46 μg·cm^−2^, whereas the content of KXL and CHL increased continuously from 2.47 to 3.02 and 0.66 to 0.89 μg·cm^−2^, respectively (Figure 2D). There were 18, 14 and 13 kinds of fatty acids explored in KXL, FTL and CHL, respectively. The most abundant fatty acid in KXL was (E)-11-eicosenoic acid, followed by (E)-9-octadecenoic acid and palmitic acid. However, the highest-dominance components of fatty acids were palmitic acid and stearic acid in FTL and (Z)-13-eicosenoic acid (C21) and (E)-13-octadecenoic acid in CHL (Figure 3). However, the main two components in KXL were not detected in FTL and CHL, and the predominant fatty acid content which remained stable during storage in CHL was undetected in KXL and CHL.

#### 3.2.5. Ketones

During storage, the levels of ketones did not change significantly, the content of KXL increased slightly and the content of FTL and CHL decreased slightly (Figure 2E). The most prominent ketone was nonacosan-10-one in the three plum cultivars, which decreased slightly during storage at room temperature.

#### 3.2.6. Aldehydes

The content of aldehydes in KXL decreased first and then increased, and in FTL it increased first and then decreased. However, the content in CHL was maintained at a stable low level during storage (Figure 2F). In total, 6, 3 and 5 types of aldehydes were detected in KXL, FTL and CHL with contents of 6.10, 1.21 and 0.19 μg·cm^−2^, respectively, at 0 d. The highest level of aldehydes was exhibited by octacosanal, accompanied by triacontanal in KXL. The aldehydes with the highest contents were octacosanal and hexacosanal in FTL and hexacosanal and heptacosanal in CHL, respectively (Figure 3).

#### 3.2.7. Esters

The content of esters decreased significantly for KXL and FTL, but it increased slightly for CHL (Figure 2G). Forty esters were identified, and pimelic acid, butyl octadecyl ester, octanoic acid, octadecyl ester and glycidyl presented the highest-dominance esters in KXL, FTL and CHL, respectively (Figure 3).

#### 3.2.8. Triterpenes

The total content of triterpenes in wax was 9.76, 4.98 and 1.99 μg·cm^−2^, accounting for 11.62%, 19.52% and 19.46% of the total wax, respectively. The content of triterpenes increased significantly after storage for 20 d at room temperature for KXL, FTL and CHL and reached 12.03, 5.64 and 2.41 μg·cm^−2^, accounting for 12.07%, 24.18% and 25.70% of the total wax, respectively (Figure 2H and Table 1). Ursolic aldehyde was the most dominant triterpene, followed by ursolic acid in KXL and FTL, and its concentration increased during storage. The ursolic acid content in CHL (1.98 μg·cm^−2^) was higher than that in KXL (1.12 μg·cm^−2^) and FTL (0.30 μg·cm^−2^), which increased during storage. However, ursolic aldehyde was not detected in CHL, and in contrast α-amyrenol was detected with trace amount of 0.01 μg·cm^−2^ (Figure 3).

#### 3.2.9. Olefins

Olefins exhibited a decline trend during storage and disappeared at 20 d, although they had a slight rise at 20 d compared to 10 d (Figure 2I). Three olefins of stigmast-5-ene, 17-pentatriacontene and squalene were screened from three plum cultivars. KXL contained three olefins, and squalene had the highest content, followed by stigmast-5-ene which was also contained in FTL, and the content of stigmast-5-ene was higher than that in KXL. However, stigmast-5-ene was not detected in CHL (Figure 3).

### 3.3. Changes in Quality of Three Plum Cultivars during Storage at Room Temperature

**Figure 4:**
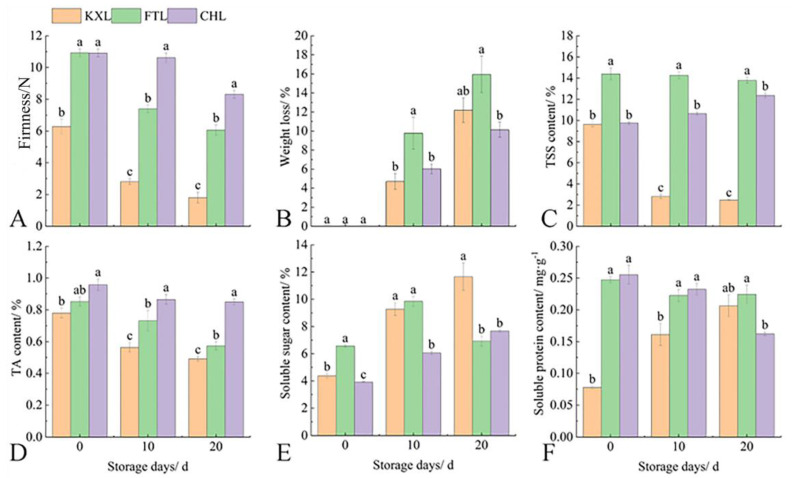
Changes in quality parameters among three plum cultivars during storage at room temperature. (**A**), Firmness; (**B**), weight loss; (**C**), TSS content; (**D**), TA content; (**E**), soluble sugar content; (**F**), soluble protein content. Note: vertical bars express the standard errors of the means (*n* = 3). Different lowercase letters indicate significant differences for each storage period (*p* < 0.05).

The firmness of KXL was the lowest (6.05 N) among the three kinds of plum, followed by CHL (10.49 N) and FTL (10.50 N) (Figure 4A). The fruit firmness of KXL, FTL and CHL decreased gradually and decreased by 71.21%, 44.53% and 23.85%, respectively, after storage for 20 d at 25 ± 1 °C (Figure 4A). The weight loss rate of FTL fruit was significantly higher than that of KXL and CHL, which reached 15.94% at 20 d, 23.59% and 36.60% higher than KXL and CHL (Figure 4B). During storage, the TSS content of KXL fruits decreased rapidly, and FTL had a slight change, while CHL increased gradually. The TSS content of FTL fruits was significantly higher than that of KXL and CHL during storage (Figure 4C). The TA content of plum fruit decreased gradually during storage, and it was 42.22% and 32.55% higher in CHL fruit than KXL and FTL at 20 d (Figure 4D). The soluble sugar content of KXL and CHL showed an increase trend during storage, while the content of FTL had a different trend from KXL and CHL, and it increased first and then decreased and reached the highest at 10 d (Figure 4E). The soluble protein content of FTL and CHL fruits decreased, while the content of CHL fruits increased (Figure 4F).

### 3.4. Cluster Analysis of Fruit Quality and Cuticular Wax of Three Plum Cultivars during Storage

**Figure 5:**
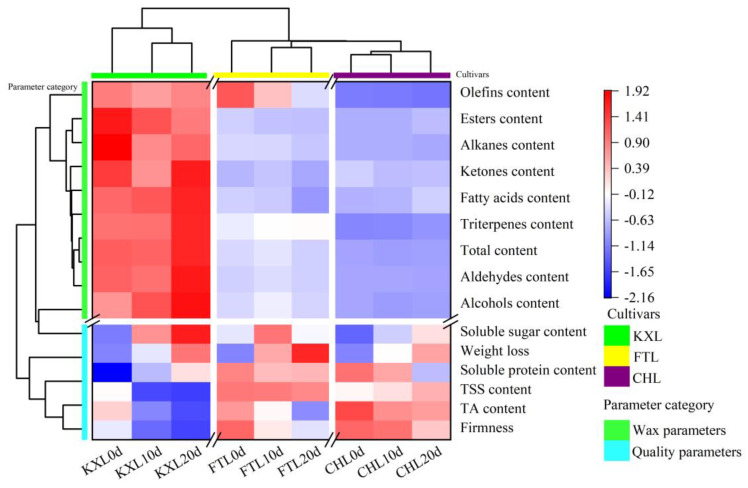
Cluster analysis of wax composition and fruit quality among three plum cultivars during storage at room temperature. Note: the color ranges from blue to red indicates low to high correlation coefficients.

In order to clarify the difference in experimental parameters of the fruit of three plum cultivars, the quality parameters and wax parameters of plum fruit were analyzed by cluster analysis. The results of cluster analysis based on samples can distinguish three plum cultivars, but the difference between FTL and CHL was not obvious. The dendrogram generated by the wax parameters and quality parameters were divided into two groups. Group 1 included nine wax parameters (alcohols, alkanes, fatty acids, ketones, aldehydes, esters, triterpenes, olefins and total content) and one quality parameter (soluble sugar content). The second group contained five quality parameters (weight loss, firmness, TSS content, TA content and soluble protein content) (Figure 5).

### 3.5. Correlation Analysis between Fruit Quality and Cuticular Wax of Three Plum Cultivars during Storage

**Figure 6:**
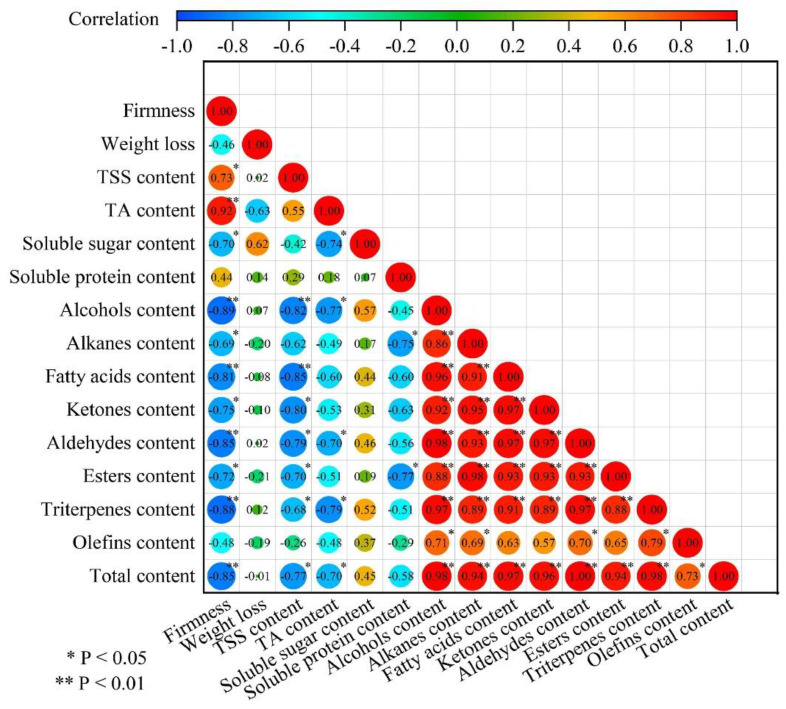
Correlation analysis between wax parameters and quality parameters among three plum cultivars during storage at room temperature. Note: the Pearson correlation coefficient is expressed by the size of the circle. Blue color indicates negative correlation, and red color indicates positive correlation. * represents significant difference (*p* < 0.05), and ** represents extremely significant difference (*p* < 0.01).

In order to clarify the correlation between quality parameters and wax parameters of three kinds of plum, the correlation between quality parameters and wax parameters of plum fruit was analyzed (Figure 6). By analyzing the quality parameters separately, the results showed that the firmness of plum fruit was significantly positively correlated with TSS content (R = 0.73, *p* < 0.05), extremely significantly positively correlated with TA content (R = 0.92, *p* < 0.01) and negatively correlated with soluble sugar content (R = −0.70, *p* < 0.05). TA content had a significant negative correlation with the content of soluble sugar (R = −0.74, *p* < 0.05). By analyzing the wax parameters separately, the results illustrated that all the wax parameters were positively correlated. Except for olefin, other wax parameters were strongly positively correlated, especially alcohols, alkanes, fatty acids, ketones, aldehydes, esters and triterpenes (*p* < 0.01).

The joint analysis of wax parameters and quality parameters revealed that the firmness of plum fruit was negatively correlated with wax parameters and was significantly negatively correlated with alcohols, triterpenes, aldehydes, fatty acids and total content (*p* < 0.01) and negatively correlated with alkanes, ketones and esters (*p* < 0.05). The weight loss of plum fruit was negatively correlated with wax parameters except triterpenes, alcohols and aldehydes, but the correlation was weak. The TSS content of plum fruit was significantly negatively correlated with wax components, alcohol and fatty acid contents (*p* < 0.01) and negatively with ketones, aldehydes, esters, triterpenes and total contents (*p* < 0.05). The TA content of plum fruit was negatively correlated with wax parameters and mainly negatively correlated with alcohol, aldehyde, triterpenes and total content (*p* < 0.05). The soluble sugar content of plum fruit was positively correlated with all wax composition, but the correlation was weak. The soluble protein content of plum fruit was negatively correlated with wax parameters and mainly negatively correlated with alkanes and esters (*p* < 0.05).

**Figure 7:**
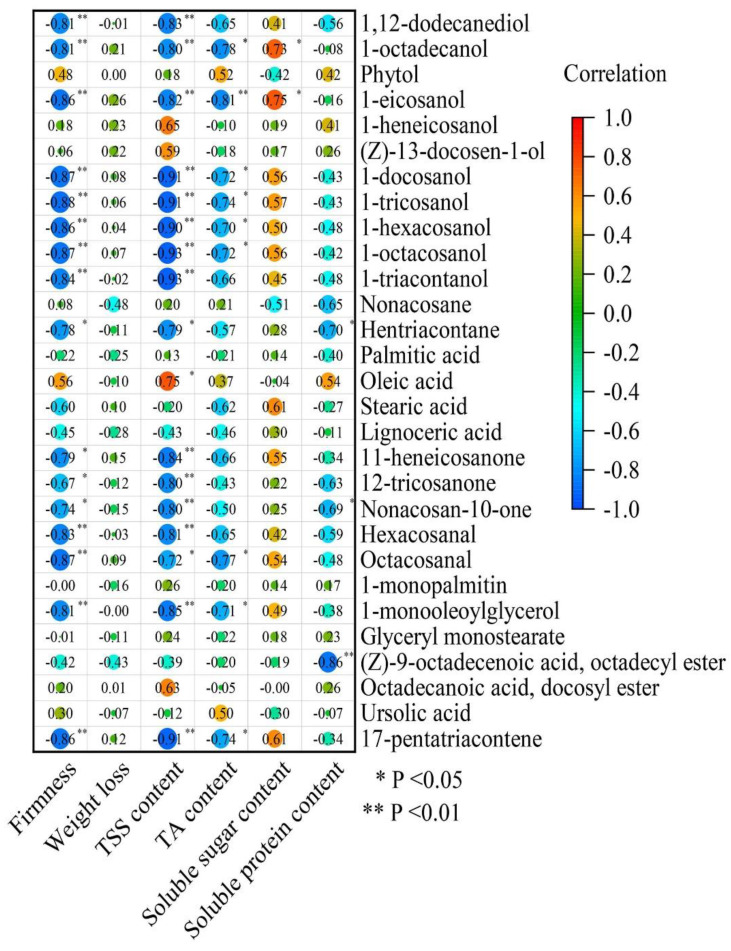
Correlation analysis between wax common constituent and fruit quality among three plum cultivars during storage at room temperature. Note: the Pearson correlation coefficient is expressed by the size of the circle. Blue color indicates negative correlation, and red color indicates positive correlation. * represents significant difference (*p* < 0.05), and ** represents extremely significant difference (*p* < 0.01).

Quality parameters of plum fruit and the common composition in three plum cultivars were analyzed (Figure 7). Most of the alcohols (1,12-dodecanediol, 1-octadecanol, 1-eicosanol, 1-docosanol, 1-tricosanol, 1-hexacosanol, 1-octacosanol and 1-triacontanol) were significantly negatively correlated with firmness, TSS and TA content. Oleic acid in the fatty acids was positively correlated with the TSS content. The 11-heneicosanone, 12-tricosanone and nonacosan-10-one of the three plum cultivars were significantly negatively correlated with firmness (*p* < 0.05) and extremely significantly negatively correlated with TSS content (*p* < 0.01). Nonacosan-10-one was also significantly negatively correlated with soluble protein content. Moreover, hexacosanal was significantly negatively associated with firmness and TSS content (*p* < 0.01). Octacosanal was extremely significantly negatively correlated with firmness (*p* < 0.01) and negatively correlated with TSS and TA content (*p* < 0.05). 1-Monoleoylglycerol was significantly correlated with firmness, TSS and TA (*p* < 0.05), and (Z)-9-octadecenoic acid and octadecyl ester were negatively correlated with soluble protein (*p* < 0.01). In addition, 17-pentatriacontene was significantly negatively correlated with firmness and TSS (*p* < 0.01) and negatively correlated with TA (*p* < 0.05).

## 4. Discussion

The cuticular wax morphology, composition and quality parameters of KXL, FTL and CHL and their relationship between wax and quality have been described. The results of our study demonstrated that the three plum cultivars contained a very prominent bloom, which can give the fruit bright color and aesthetic quality, which are among the most prominent elements attracting consumer demand for fruit. Moreover, it has a protective function against biotic and abiotic stress [4].

### 4.1. Cuticular Wax of Three Plum Cultivars

#### 4.1.1. Structure of Cuticular Wax of Three Plum Cultivars

Wax crystals have a wide range of structure types, including tubular-shaped in blueberries [16], platelet-shaped in pears [11], flattened platelets in apple fruit [8] and citrus fruit [32] and granule-shaped crystals in papaya [18]. The results of our experiment revealed that there was also a wax crystal diversity in the surface of plum, and the wax of plum fruit was composed of a mixture of rod-shaped and platelet-shaped wax crystals. KXL contained a large amount of flaky wax, FTL had a large number of granular wax crystals as well as some rod-shaped wax crystals and CHL possessed rod-shaped wax, which finally formed a large gap. At 0 d, KXL showed the highest integrity and fewer folds and cracks in the cuticular wax surface than the other two kinds of plum, in accordance with the highest content of the wax among three cultivars (Figure 1). Cuticular wax has dense molecular packing, which has been demonstrated in an assortment of other species [16,33,34,35].

During storage, there were almost no cracks in the wax of KXL, which was possibly related to the high content of wax in the pericarp of KXL and a large number of flakes of oil droplets (Figure 1). The platelet-shaped wax crystals covered with rod-shaped wax crystals of FTL gradually decreased or even partially disappeared, while rod-shaped wax crystals decreased and adhered, which was consistent with the conclusion that the characteristic tubular wax structure on blueberry surface gradually forms block adhesion during storage at room temperature [28] (Figure 1). Ding et al. [36] also demonstrated that the crystals on the surface of fruits degraded and decreased with prolonged storage time. The platelet-shaped wax crystals of the CHL gradually increased and finally formed a folded flake structure (Figure 1). Interestingly, the cracks in the wax surface of CHL fruit surface disappeared, and the surface became smooth after 20 d of storage, which was consistent with some previous studies [37,38] demonstrating that the presence of cracks in the wax surface and the specific melting of the wax at higher temperatures cover the cracks of the fruit surface and play a crucial role in maintaining fruit quality and controlling water loss [37,39].

The wax compounds of self-assembly can contribute to changing three-dimensional structures of wax crystals or the arrangement on the fruit surface, thereby leading to a transformation of wax morphology and appearance [33,40]. The crystal structure of the wax is usually determined by the crystalline compounds of alkanes and alcohols [33]. During storage, the quantity of alkanes of three plum cultivars significantly decreased and led to the degradation and reduction in the epicuticular wax crystals, which was in agreement with the results of Ding et al. [36]. However, the content of alcohols increased for KXL and decreased for FTL and CHL, and KXL had higher alkane contents than FTL and CHL, which may be the main reason for the flaky wax crystal structure of KXL. Wax crystals of most oranges are platelet-shaped, where alkanes and aldehydes are essential substances for the formation of platelet-shaped wax crystals [32,36]. A high proportion of alkanes and aldehydes are closely associated with platelet-shaped wax crystals, which is consistent with the results of our study. However, our findings were not completely in agreement with the results of Mukhtar et al. [4], where the wax platelets of European plum were formed by crystals of linoleic and ursolic acids.

#### 4.1.2. Composition of Cuticular Wax of Three Plum Cultivars

The wax synthesis pathway changes in the case of environmental stress, which contributes to the change in the final synthetic substances, thereby resulting in the differences in wax composition and content [41]. Our results also illustrated that different plum cultivars contained different wax components and their proportions of the total wax, while there were some wax components that were not detected in all plum cultivars.

Experimental results of this research demonstrated that the surfaces of KXL had the highest total wax content, CHL presented the lowest total wax amount, the change trends of the cuticular wax content of different plum cultivars were different and the cuticular wax content of KXL increased during storage, while that of FTL and CHL decreased (Figure 2). A similar phenomenon of cultivar-dependent variability was also observed in apple [12,19,42], citrus [32] and bilberry [43] fruits. The differences in varying tendency of wax during storage may be ascribed to cultivars [9,12,28], development and storage conditions [11,36,44] as well as some preservative treatments [29,42,45].

In the present study, the surface waxes of the plum at harvest and storage fruit were mainly composed of alcohols, alkanes and triterpenes. The distribution density of flaky wax structure in plum peel was closely related to the contents of alkanes and alcohols. Alkanes, especially nonacosane, decreased during storage (Figure 3), which was consistent with previous studies including apples [12,39,42], citrus [32], grapes [46] and pears [11]. The content of nonacosane can maintain the three-dimensional structure of wax, and with a decrease in its content the structure changes, which prominently resulted in the softening of plums. It was speculated that nonacosane also played an important part in maintaining the three-dimensional structure of plum cuticular wax. Alcohols could be assigned as the highest-content components in plum cuticular wax and played an important role in the formation of wax. Alcohol content increased in KXL but decreased in FTL and CHL, which was consistent with the trend of total wax content (Figure 2). Interestingly, 10-nonadecanol was only present in KXL, and 1-triacontanol and 1-hexancosanol had higher content in KXL than FTL and CHL (Figure 3), which may have led to KXL having poorer quality than the other two plum cultivars. It was reported that nonacosan-10-ol resulted in increased greasiness and worse quality of Jonagold apple [39]. Nonacosan-10-ol also played a critical role in maintaining the three-dimensional structure in the apple wax and led to apple greasiness [42,47]. However, nonacosane-10-ol was absent in plum fruit, which may be part of the reason for plums without greasiness.

Triterpenes were also important substances, accounting for 11.62%, 19.52% and 19.46% of total wax in three plum fruits, which increased steadily during storage (Table 1). An increase in triterpenes has been observed in blueberry [29] and sweet cherry [48]. The most obvious difference among triterpenes was the complete absence of ursolic aldehyde in the wax of CHL, while it was one of the predominant components for KXL and FTL, with a higher concentration for KXL (Figure 3). Ursolic acid was present in three kinds of plum and increased during storage, which was in agreement with the findings of Chu et al. [10] in blueberries. Surprisingly, α-amyrenol and betulin were only present in CHL although with a trace amount and decreased amount during storage (Figure 3). It is noted that α-amyrenol, with anti-inflammation and anti-aging effects, has been observed in citrus [32], pears [28] and Pingguoli pears [49]. Betulin has attracted much attention because of its wide range of biological activities in inhibition of melanin, reduction in blood fat, protection of the liver and its anticancer, antitumor and antivirus properties, and it is widely utilized in the medicine, food and cosmetics industries [35]. Fatty acids and aldehydes were also included in the wax composition. The esters in fruit wax were mainly composed of long-chain fatty acid methyl esters and long-chain fatty acid triglycerides (Figure 2 and Table 1), which were also demonstrated in blueberry [29] and pear [28].

### 4.2. Fruit Quality of Three Plum Cultivars

For those three plum cultivars, firmness and TA decreased, and soluble sugar increased, although with a different change trend. TSS decreased for KXL and FTL and increased for CHL, but the content of TSS was higher than CHL. Overall, the TSS decreased. Therefore, the firmness of plum fruit was significantly positively correlated with TSS content TA content (Figure 7), which has been illustrated in grape [45] and apricot [50]. There was a negative correlation between firmness and soluble sugar content, which was consistent with the findings of Lin et al. [26]. Weight loss had a negative correlation with TA content and positive correlation with the soluble sugar content, which showed that weight loss and soluble sugar increased as TA was consumed as a respiratory substrate [45]. TSS content had a negative correlation with the content of soluble sugar. The experimental result revealed that the sugar was classed in group 1, and the TSS was in group 2 (Figure 5). Moreover, soluble sugars included sucrose, glucose, fructose, sorbitol and a small amount of inositol. Zhao et al. [51] demonstrated that sucrose and sorbitol decreased while glucose and fructose increased with the increase in storage time. TSS was negatively related to soluble sugar, possibly due to a faster decrease in the content of soluble sugars such as glucose and fructose [51].

### 4.3. Relationship between Cuticular Wax and Quality Parameters

A growing number of studies have recently illustrated that cuticular wax, primarily structure and composition, has a considerable effect on the postharvest storage quality of horticultural products with regard to its role as an obstruction to combat damage [5,13,16]. Our results indicated that there was a close correlation between wax structure, composition and weight loss, as well as postharvest softening and quality retention.

#### 4.3.1. Cuticular Wax Structure and Quality Parameters

The wax crystal structure plays a vital role in preventing water loss. López-Castaňeda et al. [52] reported that the wax crystals in the form of flakes or plates usually lead to higher water loss, whereas the threadlike or tubular wax crystals contribute to water retention, and the granule-shaped structure of a crystal has a positive effect on weight loss. In our study, FTL had the highest weight loss after storage for 20 d, which may be due to the granule-shaped wax crystals, although the wax crystals of KXL were flaky, which led to a lower weight loss than FTL, possibly because of smaller holes in the epidermis of the surface. Yang et al. [34] found that the wax disintegration of apple peel would lead to a decrease in enthalpy of wax, while a decrease in enthalpy of wax may have resulted from the wax disintegration of KXL peel at room temperature protecting water loss and maintaining fruit quality. The wax structure was destroyed during storage, which led to a higher weight loss rate, accelerated physiological metabolism and a decrease in firmness [11,29].

#### 4.3.2. Cuticular Wax Composition and Quality Parameters

##### Cuticular Wax Composition and Weight Loss

Our experimental results manifested that total wax and wax components were all weakly correlated to weight loss in three plum cultivars. Total wax, alkanes, fatty acids, ketones, esters and olefins had a negative correlation, and triterpenens, alcohols and aldehydes had a positive correlation (Figure 6 and Figure 7).

The research results demonstrated that the alkane content was negatively correlated with the weight loss of three plum species. Alkanes, as the main component of wax, are beneficial to maintaining fruit moisture [53] and have stronger resistance to water movement than other components in the wax. The wax crystals with a flat structure are formed on the peel of the fruit, and molecular crystals with terminal polar groups form a double-layer knot with a head-to-head orientation [54]. This double-layer structure of low polar components wraps the fruit. The low polar components more effectively prevent water loss due to their powerful water repellency. Compared with other components of the same carbon chain, alkanes represented the lowest polarity and are more conducive to fruit water retention. In addition, alkanes respond to water stress and enhance the cuticle barrier characteristics of *Arabidopsis* leaves [55], and their weighted average chain length is negatively correlated with water permeability [56]. The alkane content was negatively correlated with the weight loss of three plum species, which was consistent with the results of Wang et al. [11] in ‘Korla’ pears at room temperature and cold storage. After 10 d at room temperature, KXL showed the highest content as well as the lowest weight loss, but at 20 d, FTL manifested the highest weight loss. FTL probably had higher TSS, and in the later storage times the consumption of nutrients dominated the weight loss (Figure 4). Moreover, nonacosane had a negative correlation with weight loss (Figure 7), and similar results were obtained in pears [11]. In addition, the amount of nonacosane may be related to cracking tolerance of sweet cherries [57].

Fatty acids can inhibit dense molecular packing of crystals in the cuticle membrane and form a looser network giving rise to water loss and softening [7,32]. Fatty acids are usually produced at higher temperatures and in decarbonylated substances at lower temperatures during storage [44]. The results of our research showed that fatty acid increased and led to higher weight loss during storage in KXL and CHL, which was consistent with previous findings [7,32]. However, fatty acid decreased in FTL, which agreed with the finding of Chu et al. [10] in blueberry.

The triterpenes were positively correlated with the weight loss rate. With prolonged storage time, the synthesis mechanism of triterpenes responds, thus promoting the increase in triterpenes content and enhancing the correlation between triterpenes and the weight loss rate. Yang et al. [5] demonstrated that triterpenes were also important components in grape inner wax that affected fruit weight loss. In our study, ursolic aldehyde had a greater impact on the weight loss of plum fruit compared with ursolic acid. However, oleanolic acid had a more obvious effect on reduction in weight loss of grape [5] and blueberry [10] than ursolic acid.

In addition to triterpenoids, alcohols and aldehydes were positively related to the weight loss rate, but the correlation was much weaker than that of triterpenes (Figure 6). Previous studies showed that wax components, especially alkanes and triterpenes, were closely correlated to water conservation [45,51]. The experimental results showed that higher contents of alkanes and lower contents of triterpenes resulted in stronger water retention capacity in plum, and similar results were obtained by Dong et al. [42] in apple fruit and Leide et al. [58] in tomato fruit, but these findings were inconsistent with those of Li et al. [45] in grape, where lower alkanes and higher triterpenes gave rise to stronger water conservation ability, and those of Jiang et al. [29] in blueberry, in that higher both alkanes and triterpenes contributed to water retention capacity.

##### Cuticular Wax Composition and Fruit Softening

In the present study, wax components except olefins were significantly negatively related to firmness in three plum cultivars, particularly alcohols, fatty acids, aldehydes and triterpenes (Figure 6). However, other studies demonstrated that there was a positive relationship between firmness and the amount of total wax and composition [45]. Li et al. [45] exhibited that alcohols and fatty acids were positively related to firmness in grapes [45], which was not consistent with our results. However, for Korla pear, fatty acids and olefins had a negative correlation and alkanes and esters had a positive correlation with firmness [11]. Fatty acids were also the most cuticular wax in MT-treated KXL fruit, which correlated significantly positively with firmness [26].

The effect of wax composition and content on fruit firmness has been demonstrated in a wide range of studies [10,13,59]. However, there was a positive relationship in single composition of oleic acid, phytol, ursolic acid, 1-heneicosanol, nonacosane and firmness (Figure 7), the existence of which can maintain a higher firmness of plum fruit. Lin et al. [26] demonstrated that waxes can affect the firmness changes due to the chemical and mechanical properties of fruit cell walls.

##### Cuticular Wax Composition and Fruit Quality

Wax components were also negatively related to TSS, TA and soluble protein content while being positively correlated with soluble sugar (Figure 6 and Figure 7), which may be the reason why soluble sugar was classified in group 1 and others in group 2 (Figure 5). Alcohols, fatty acids, ketones and aldehydes were related to fruit quality. For example, alcohols, including 1,12-dodecanediol, 1-octadecanol, 1-eicosanol, 1-docosanol, 1-tricosanol, 1-hexacosanol, 1-octacosanol and 1-triacontanol and 1,12-dodecanediol, 11-heneicosanone, 12-tricosanone and nonacosan-10-one, octacosanal, hexacosanal, 1-monooleoylglycerol and 17-pentatriacontene, in the wax were negatively correlated with TSS and TA, positively correlated with soluble sugar content, effectively delayed the senescence of plum fruit and was conducive to preservation of the TSS and TA content in the fruit during storage (Figure 7). Oleic acid was positively correlated with the TSS and TA content and positive regulation of soluble sugar content changes and may have contributed to the decrease in the fruit softening and consequently affecting the storage quality of the plum fruits [11]. (Z)-9-octadecenoic acid, octadecyl ester, 1,12-decanediol, nonacosane, hentriacontane and nonacosan-10-one in plum wax could effectively inhibit the increase in soluble protein content in plum fruit. Nonacosan-10-one was the dominant ketone in three plum cultivars, and it was reported that nonacosan-10-one is related to fruit quality deterioration [42,56].

All in all, it is not a single component but a combination of multiple components that determine the fruit quality. Chu et al. [16] found that the contents of alkanes, primary alcohols and fatty acids jointly determine the weight loss of blueberry fruit. Alkanes and triterpenes have been found to control water penetration in the cuticle of citrus fruit [3]. Alkanes and aldehydes can regulate the water balance. The wax can inhibit fruit quality deterioration by changing its composition and distribution or by coverage in cracks and stomata [60]. However, how to determine the fruit quality among different components together still needs further research, especially the molecular mechanism.

## 5. Conclusions

In summary, the wax crystal structure and chemical composition as well as the relationship between wax and storage quality in three plum cultivars were investigated. (1) The differentiation of cuticular wax morphology and composition was significantly cultivar-dependent. The surfaces of three plum cultivars were composed of different degrees of rod-shaped wax and platelet-shaped wax, which degraded and melted after storage. The crystal structure was different among three plum cultivars, which can be partially ascribed to the different proportions of wax components. (2) Although the wax concentration and compounds varied drastically among cultivars, alcohols, alkanes and triterpenes were the predominant wax compounds in three plum cultivars, with a decrease in alkanes and an increase in triterpenes content during storage; however, a decrease in alcohols for FTL and CHL and an increase in alcohols for KXL were observed. (3) Alcohols, triterpenes, fatty acids and aldehydes were most dramatically correlated with the senescence of fruit, and alkanes, esters and olefins were most significantly correlated with water loss. Nonacosane and ursolic aldehyde can enhance the water retention of fruit. The main components with the highest contents in the three plum cultivars were nonacosane, 1-triacontanol, 1-heneicosanol, nonacosan-10-one, octacosanal, ursolic aldehyde and oleic acid. (4) The results not only provide an overview of cuticular wax of plum but also explore how wax components change during plum storage and how wax components may contribute to postharvest storage quality of plums and will provide a theoretical reference for the further precise development of edible plum fruit wax.

## Figures and Tables

**Table 1:** Changes in proportion of cuticular wax composition among three plum cultivars during storage at room temperature.

Cultivars		KXL			FTL			CHL		
	Storage Days/d	0	10	20	0	10	20	0	10	20
Content/%										
Alkanes		34.61	21.64	24.97	7.24	7.06	5.53	3.19	3.36	2.82
Alcohols		38.74	51.76	52.60	44.54	49.40	45.65	39.53	27.64	30.37
Fatty acids		2.94	3.16	3.03	3.50	2.99	2.00	6.50	7.91	9.58
Ketones		1.26	1.00	1.15	1.27	1.24	1.22	3.87	3.90	3.75
Aldehydes		7.27	7.02	7.97	4.76	5.03	5.17	1.87	1.74	1.29
Esters		3.41	2.91	2.03	2.05	1.41	1.69	2.44	2.89	3.96
Triterpenes		11.62	11.92	12.07	19.52	19.14	24.18	19.46	23.16	25.70
Olefins		0.14	0.13	0.12	0.54	0.32	0.21	0.08	0.08	0.04

## Data Availability

Data are presented within the article.
